# Isoprenaline in complete heart block patients during ambulance transport

**DOI:** 10.1186/1865-1380-8-S1-P3

**Published:** 2015-04-22

**Authors:** Taraknath Taraphdhar, Sreekanth Reddy Basireddy

**Affiliations:** 1 The Mission Hospital, Durgapur, West Bengal, India

## Objectives

Isoprenaline has been recommended for short term treatment of heart block by the World Health Organization (2004), Australasian Resuscitation Council (2009), and UK Resuscitation Council (2010) guidelines. This study aims to ascertain the role of Isoprenaline in management of complete heart block during ambulance transport.

## Methods

Retrospective study based on medical records of adult patients with documented history of Complete Heart Block and transported by ambulance of The Mission Hospital, Durgapur, from January 2011 to June 2014. Data was analyzed by simple statistical methods.

## Results

98 (69 male and 29 female) such patients were transported, and 13 were on Temporary Pacemakers. 97 were inter-hospital transfers. 43 (44.3%) patients were transported with Isoprenaline (average dose 2.7 mcg/min, range 1 to 8.2mcg/min); 8 with Dopamine, and 7 on external cardiac pacing. 30 patients were on Isoprenaline and 8 on Dopamine in previous hospitals, and the same drug with similar dose was initially continued in the ambulance.

Out of the 84 patients transported without temporary pacemaker, 60 patients were transported within 20 km (travel time less than 30 minutes), 22 from distance between 50 to 100 km (average travel time 90 minutes) and 3 patients were transported beyond 100kms. The longest distance patients travelled with Isoprenaline infusion was 525 km (travel time 26 hours). 6 (14%) patients with Isoprenaline showed side effects and dose had to be reduced. Isoprenaline was replaced by Dopamine in one of them. 4 of them travelled distance between 60 to 70 km and 2 beyond 100 km. 34 (79%) patients with Isoprenaline achieved heart rates above 60/min.

## Limitation

This study is based on only one EMS operator and primarily on inter-hospital transfer.

## Conclusion

Isoprenaline can be an effective and safe drug for transport of complete heart block patients over short distances. Further study is required for their safety in long distance travel.

